# Micromachining of Transparent Biocompatible Polymers Applied in Medicine Using Bursts of Femtosecond Laser Pulses

**DOI:** 10.3390/mi11121093

**Published:** 2020-12-10

**Authors:** Evaldas Kažukauskas, Simas Butkus, Piotr Tokarski, Vytautas Jukna, Martynas Barkauskas, Valdas Sirutkaitis

**Affiliations:** 1 Laser Research Center, Faculty of Physics, Vilnius University, Saulėtekio Ave. 10, LT-10223 Vilnius, Lithuania; simas.butkus@ff.vu.lt (S.B.); vytautas.jukna@ff.vu.lt (V.J.); martynas.barkauskas@ff.vu.lt (M.B.); valdas.sirutkaitis@ff.vu.lt (V.S.); 2 Msl Med Services Ltd., Rodou 6, Tremithousa, Paphos 8270, Cyprus; tokarski.ils.germany@gmail.com; 3 Light Conversion, Keramikų 2b, LT-10233 Vilnius, Lithuania

**Keywords:** femtosecond micromachining, burst processing, intraocular lens, hydrophilic acrylic, surface roughness, polishing

## Abstract

Biocompatible polymers are used for many different purposes (catheters, artificial heart components, dentistry products, etc.). An important field for biocompatible polymers is the production of vision implants known as intraocular lenses or custom-shape contact lenses. Typically, curved surfaces are manufactured by mechanical means such as milling, turning or lathe cutting. The 2.5 D objects/surfaces can also be manufactured by means of laser micromachining; however, due to the nature of light–matter interaction, it is difficult to produce a surface finish with surface roughness values lower than ~1 µm Ra. Therefore, laser micromachining alone can’t produce the final parts with optical-grade quality. Laser machined surfaces may be polished via mechanical methods; however, the process may take up to several days, which makes the production of implants economically challenging. The aim of this study is the investigation of the polishing capabilities of rough (~1 µm Ra) hydrophilic acrylic surfaces using bursts of femtosecond laser pulses. By changing different laser parameters, it was possible to find a regime where the surface roughness can be minimized to 18 nm Ra, while the polishing of the entire part takes a matter of seconds. The produced surface demonstrates a transparent appearance and the process shows great promise towards commercial fabrication of low surface roughness custom-shape optics.

## 1. Introduction

Vision is one of the most important senses, which enables us to experience and understand our surroundings. However, the eye, just as any other organ or tissue in the human body, deteriorates with age and vision deterioration substantially contributes to the decrease in the quality of life. According to statistics, about 50% of Americans have developed cataracts by the age 75–79 [1] and every third person in the world suffers from myopia [2]. Today’s advanced technologies provide means to cure the above-mentioned eye conditions by changing the natural lens of the eye with vision implant known as intraocular lenses (IOL). However, the manufacturing of intraocular lenses remains time consuming and an economically challenging procedure to this day, this is especially true when considering customized IOLs.

Intraocular lenses are typically manufactured using lathe cutting, milling, compression molding or injection molding [3]. All of the above-mentioned methods use mechanical tools that have their downsides. Diamond tools used for lathe cutting are expensive, need constant recalibration and wear out with time; therefore, leading to the increase of the surface roughness of the produced part. In addition, milling of the lens contour is a time intensive process taking up to five minutes per lens, the calibration of the equipment can take up to two hours daily [4]. Other methods that include melting of plastic and then molding are more suitable for mass production as they allow production of large quantities of units in a short period of time. Even though molding is great for mass production, this technology is not suited for the production of customized lenses, as every customized lens requires a new custom mold. The manufacturing of molds requires high precision machinery; therefore, the molds are expensive and difficult to manufacture [5]. Despite which method is used for the manufacturing of the lenses, the surfaces typically do not satisfy optical-quality standards and require an additional polishing procedure to be carried out. Both sides of the lens need to be polished in order to reach optical-quality standards (<10 nm Ra). Polishing methods such as pitch polishing, polishing using synthetic pads or magneto rheological figuring [6] are not suitable for intraocular lens production as there is a high risk of damaging the lens haptics. To this day all intraocular lenses are polished using the tumbling method, where lenses are tumbled in a mixture of glass beads, alcohol and deionized water. This method is capable of producing surfaces of superb quality, in other words, Ra in the range of a few nanometers [7]; however, the tumbling process may take as long as few days [8]. To avoid the stated disadvantages of mechanical manufacturing, laser micromachining could be used as an alternative to mechanical manufacturing means of intraocular or other type of optical lenses. Nowadays it is possible to achieve high ablation efficiencies of 1.23 µm/pulse using Ti:Sapphire laser pulses of 30 µJ energy in polymer poly(methyl methacrylate), better known as PMMA [9]. In addition, the surface quality achieved may be in the range of a few hundred nm to ~1 µm depending on wavelength used [10]. This is achieved thanks to the advanced technologies of ultrashort pulse generation in the pico and femto duration range. The peak intensity of focused ultrashort pulses can be as high as 10^13^ W/cm^2^ enabling the initiation of non-linear processes such as multi-photon absorption and avalanche ionization that enable the micromachining of materials that under normal circumstances (visible to infrared spectrum) are transparent. When using femtosecond laser pulses the material is instantly vaporized, resulting in relatively ”cold” ablation regimes without producing heat-affected zones (also known as HAZ) [11]. The utilization of ultrashort infrared femtosecond pulses in the field of laser micromachining provides the flexibility to produce almost any shape from transparent or non-transparent materials. However, the non-linear absorption of the pulse and accompanied instant material evaporation alone is not sufficient to produce an optical-grade quality surface finish resulting in surface roughness >1 µm Ra. Transparent surfaces with roughness of >1 µm exhibit strong light scattering and may reduce imaging quality of these components to such a degree that it becomes too low for optical applications. Surface roughness is also important for the bio-compatibility of the implants [12,13,14]. Rough surfaces on the implants can damage human tissues or be rejected by the immune system. Moreover, it was seen that implants with higher surface roughness values increase the risk of post–surgical complications (e.g., posterior capsular opacification) that lead to decrease in vision [15]. Recently it was discovered that using bursts of femtosecond laser pulses, when each pulse is divided into a sequence of sub-pulses with a temporal separation of few tens of nanoseconds or hundreds picoseconds, improves laser material processing by boosting the ablation efficiency [16,17,18,19,20,21]. It was also demonstrated, that by using bursts of pulses it is possible to further decrease the surface roughness compared to using the conventional single femtosecond pulse regimes [22].

The aim of this study is the investigation of the polishing capabilities of rough (~1 µm Ra) hydrophilic acrylic (typical material used for vision implants) surfaces using bursts of femtosecond laser pulses, while the initial rough surface of the samples was prepared by femtosecond laser ablation to a desired shape without using bursts. It was shown, that by tailoring the properties of the burst (the shape of the envelope, number of pulses, average power, etc.) it is possible to control the amount of heat flux entering the material and in such a way realize a controlled melting procedure that reduces the surface roughness from ~1 µm Ra to <20 nm Ra. These results show great potential for the industrial scale production of customized optical components made from transparent, biocompatible polymer materials.

## 2. Materials and Methods

**Figure 1:**
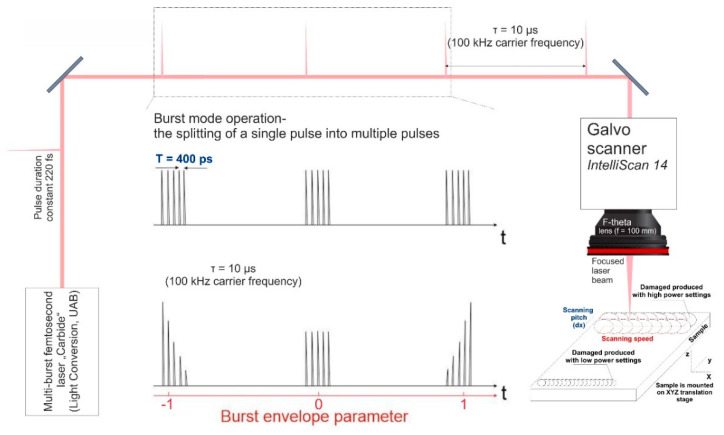
Micromachining setup used for ablation and subsequent polishing of plastic materials. A single pulse may be split into a sequence of sub-pulses (bursts), the temporal separation between each sub-pulse within the burst was constant at 400 ps. Additionally, by varying the burst envelope parameter it is possible to adjust the energy distribution within the burst.

In this work we used a multi-burst femtosecond laser “Carbide” (Light Conversion, Vilnius, Lithuania). With a central wavelength of 1030 nm, generating pulses of 220 fs (full width at half maximum) duration at a repetition rate of 100 kHz, with a maximum average power of 40 W. The laser is able to produce controllable bursts of pulses, meaning that each pulse may be divided into a sequence of sub-pulses with a variable energy distributed through the sub-pulses as shown in Figure 1. The temporal separation between the sub-pulses may be set to either 400 ps or 15.5 ns. In the scope of this article, only the temporal separation of 400 ps was investigated. The formation of femtosecond bursts is done by storing pulses in the regenerative amplifier and controllably expelling them. The control of the expelling pulse energy is done by using electro-optical switches. The precise timing of the round trip of the regenerative amplifier and the time delay between the pulses from the master oscillator is crucial [23]. In addition, the laser has a built-in software feature that allows tailoring of the energy distribution of the sub-pulses within the burst which can produce an ascending or descending burst amplitude envelope, meaning it is possible to form a rising/falling energy envelope, see Figure 1. To characterize the energy of the sub-pulses within the burst we used the “Time-correlated Single Photon Counting” (also known as TCSPC) method [24] and MATLAB (R2018a version) for data visualization. The micromachining of the material was done by focusing the beam via a telecentric 100 mm F-theta lens, and controlling the position of the beam on the surface of the sample using a galvanoscanner “intelliSCAN 14” (SCANLAB, Munich, Germany), the setup is shown in Figure 1. In addition, the scanner with the lens was mounted on a z-axis translation stage, thus enabling the manipulation of the beam in x, y and z directions. The position of the beam on the sample was controlled using micromachining software DMC (1.4.71 version). The sample was precisely positioned below the scanner using a firmly fixed custom-built sample holder. The samples were Contamac CONTAFLEX 26% UV-IOL (R) hydrophilic acrylic tablets having diameter of 15 mm and thickness of 3 mm. The beam waist was positioned directly on the surface of the sample and the focal position was fine-tuned by firing single pulses at the sample’s surface at different heights with a step of 100 µm until the smallest diameter crater was achieved. The produced craters were investigated using an “Olympus BX51” (OLYMPUS, Hamburg, Germany) microscope. We determined that using an average laser power of 2.5 W (fluence—6.5 J/${\mathrm{cm}}^{2})$ the diameter of the crater at the focal position was 20.7 µm, while using an average laser power of 33.6 W (fluence—88 J/${\mathrm{cm}}^{2}$) the diameter of the crater was 77.3 µm. To determine the material ablation threshold we used a method described in [25] that consists of several steps: first, the square of the ablated crater diameter is plotted versus the logarithm of the pulse energy. From the slope of the distribution the focal spot $\omega_{0}\left(1/e^{2}\right)$ can be calculated. Having the spot size, the fluence was calculated and crater diameter squared versus the logarithm of fluence was plotted. By extrapolating the dependence to zero the ablation threshold was determined and was found to be 2.7 J/${\mathrm{cm}}^{2}$.

The experiment consisted mainly of two parts: first-characterization of the burst using the TCSPC setup and second-analysis of the micromachined sample. We used the characterization of the burst pulses to find the correct settings of the laser so that each sub-pulse in the burst would have the same energy. To achieve that we placed a scattering surface at an angle of 45° obstructing the beam and collecting the scattered light using a semiconductor detector “ID100-20 visible Single-photon detector” (ID QUANTIQUE, Geneva, Switzerland) and a multichannel electronic system “PicoHarp 300” (PicoQuant, Berlin, Germany). The bursts were registered and visualized using data visualization software. The temporal resolution of the imaging was 50 ps, which enables the accurate representation of the energies of the sub-pulses within the burst. The scattering surface was necessary for decreasing the intensity of the laser beam so as not to damage the detector and avoid possible reflections from the optical surfaces that could influence the registered signals. The parameter that controls the burst energy envelope was changed from −1 to 1 (see different value meanings in Figure 1) for different burst packets consisting of 2, 5, 10 and 25 sub-pulses per burst. By changing the burst envelope parameter, different configurations of sub-pulse amplitudes were obtained and analyzed.

**Figure 2:**
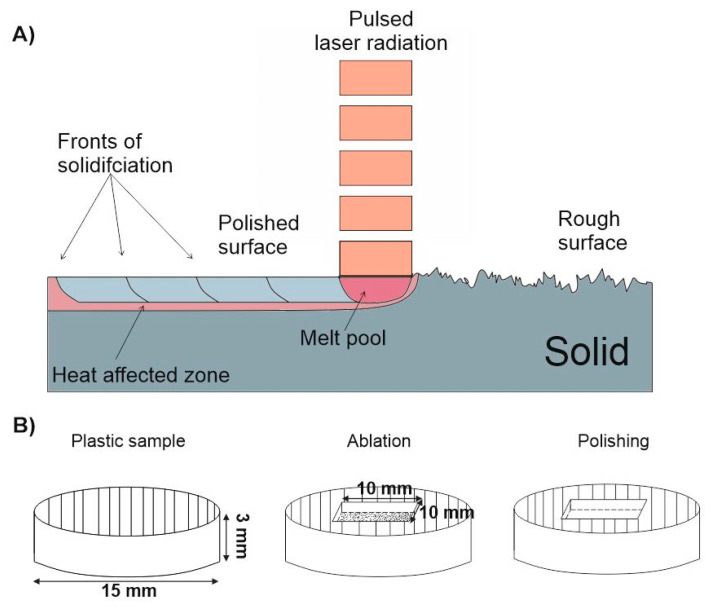
Laser polishing using pulsed laser radiation scheme (**A**), micromachining steps used for polishing (**B**). The structure of 10 mm × 10 mm × 0.5 mm was ablated and then polished using bursts of femtosecond pulses.

Micromachining of plastic experiments were carried out using a burst envelope parameter value at which all sub-pulses had the same energy. A cuboid having dimensions of 10 mm × 10 mm × 0.5 mm (such dimensions are typical for eye implants) was ablated using the conventional femtosecond ablation regime (single pulses) when the laser was operating at 100 kHz. For ablation of the surface, the overlap of beams was kept constant at 50% in both x and y directions to ensure uniform surface modification over the whole area. After the ablation step a series of experiments were performed, where combinations of bursts with different parameter settings were used for polishing of the ablated surface, see Figure 2B. In the framework of this article, surface polishing is regarded as a sensitive thermodynamical process that is based on remelting of a thin surface layer. While the material is in the liquid state it is pulled in the directions where the surface tensile force is strongest, meaning that it is pulled mainly into various valleys and cavities, hence the surface roughness after solidification may get smaller if the correct laser heating parameters are found. In addition, this process results in a combination of remelting of a thin surface layer smaller than 5 µm and vaporization of micro edges [26,27,28,29,30], see Figure 2A. Using laser radiation, it is possible to enhance the surface quality of many different materials such as metals, plastics, ceramics, glasses, etc. Hence, laser polishing is a highly used technique for industrial applications [31,32,33].

In our study the surface polishing experiments were conducted using a galvo-scanner based scanning/focusing system. By changing the average laser power (P), the scanning velocity of the beam on the surface of the sample (v), the line scanning pitch (dx) and the number of sub-pulses within the burst ($N_{p})$, the polishing capabilities of the plastic material were investigated in order to achieve the best surface quality. While conducting micromachining of samples, the temperature at the surface was monitored using a thermal vision camera “FLIR A600-Series” (FLIR systems, Hoogstraten, Belgium); for image and video processing we used the “FLIR ResearchIR” (ResearchIR MAX 4 version) software package. We also examined how the initial surface roughness impacts the polishing process. Therefore, we conducted a parametric study, where multiple samples were ablated with different settings so as to produce a different surface finish (roughness values ranging from 0.7 to 3 µm) and evaluated its roughness after polishing. In addition, a sample having roughness of Ra = 40 nm, which was prepared by mechanical polishing, was also studied. The “Sensofar PLU 2300” optical profiler (SENSOFAR, Terrassa, Spain) was used for the investigation of the surface topography while the bright field images were taken with the “Olympus BX51” (OLYMPUS, Hamburg, Germany) (10× magnification) and “Olympus LEXT OLS5000” (10× magnification) microscopes (OLYMPUS, Hamburg, Germany). The roughness parameter $R_{a}$ (arithmetic average height) was chosen as an evaluation criterion of the surface roughness. It is defined as an average absolute deviation of the height from the mean line over one sampling length [34] (in our case sampling length was 125 µm). To process the profiler data, “SensoMap” software was used.

## 3. Results and Discussion

### 3.1. Burst Investigation Results

**Figure 3:**
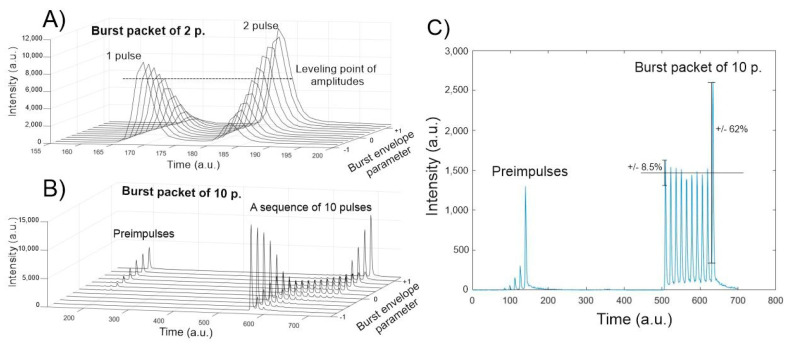
The dependence of the intensity of the sub-pulses in the burst versus the envelope parameter when a single pulse is divided into 2 sub-pulses (**A**) and into 10 sub-pulses (**B**). Amplitudes of a leveled burst packet of 10 sub-pulses are displayed in (**C**).

Measuring the individual amplitudes of each sub-pulse within the burst is complicated with conventional photodiodes and oscilloscopes due to the insufficient temporal resolution; therefore, the TCSPC method was used. The main reason for these measurements was to adjust the burst envelope parameter in such a way that all of the energies of the sub-pulses within the burst are equal or as close to equal as possible. It was found that tailoring of the burst envelope parameter was necessary to produce a flat envelope for a different number of sub-pulses within the burst. Knowing the individual energy of the sub-pulses is important for experiment reproducibility and for comparison purposes with different burst settings. The experiments were carried out by dividing one pulse into packets of 2, 5, 10 and 25 sub-pulses and different sub-pulse amplitudes were produced when changing the burst envelope parameter. It was determined that splitting a single pulse into a burst is done efficiently up to 10 sub-pulses. Splitting the pulse into more than 10 sub-pulses causes formation of parasitic pre-pulses, see Figure 3B,C. The true origin of the pre-pulses remains unknown, as it is a direct cause of the laser construction. Analysis of the measurement data showed that it is possible to level all sub-pulses within the burst to the same energy with an error of 10% except for the last sub-pulse. However, if the number of sub-pulses within the burst is lower than five, then all of the pulses within the burst are leveled to an 10% error margin. If the number of pulses is greater than five, then the trailing sub-pulse has a higher energy compared to the other sub-pulses (the more sub-pulses in the burst, the higher the energy of the last sub-pulse), this phenomenon is an inherent laser feature. Another interesting insight is that the burst envelope parameter takes on different values for a different number of sub-pulses. It was expected, that the burst envelope parameter value of 0 would yield a burst with equal sub-pulse amplitudes; however, it was found that the envelope appears to be slightly shifted towards an ascending shape (amplitudes of the sub-pulses are increasing). When performing polishing experiments, the burst envelope parameter was varied for the different cases in order to produce a flat envelope.

### 3.2. Plastic Ablation Results and Polishing Capabilities of Bursts of Femtosecond Laser Pulses

**Figure 4:**
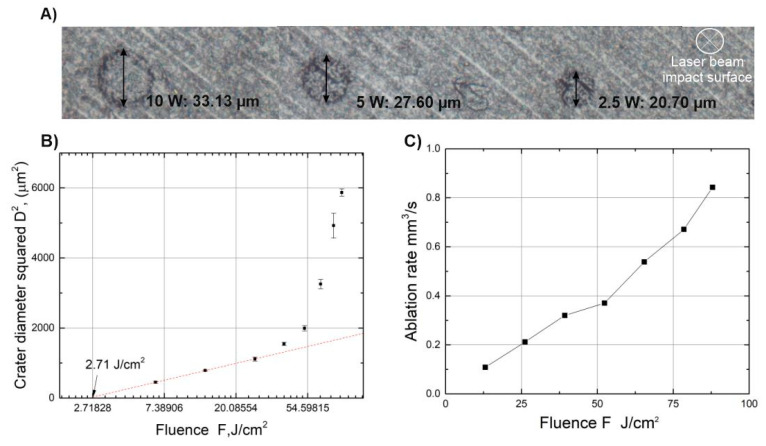
Bright field images of ablated craters taken with “Olympus BX51” microscope (**A**). Crater diameter squared dependence on fluence for ablation threshold retrieval (**B**). Ablation rate dependence on beam fluence (**C**).

Different materials ablated with femtosecond pulses is not a new topic, therefore an investigation regarding the ablation efficiency when working with different laser parameters was not performed. The preparation of the samples was done with the same laser system in single pulse mode. The ablation threshold was determined for hydrophilic acrylic using the method described in the previous chapter and was found to be 2.7 J/${\mathrm{cm}}^{2}$, see Figure 4B. The determined ablation threshold agrees with the results published by other parties when similar materials were investigated. Nam et.al. [35] and Baudach et.al. [36] reported thresholds of 2.4 J/${\mathrm{cm}}^{2}$ and 2.6 J/${\mathrm{cm}}^{2},$ respectively, while using Ti:Sapphire laser of λ = 800 nm wavelength and pulse duration of τ = 150 fs. On the other hand, Heberle et.al. [10] reported ablation thresholds of Contaflex yellow and Contaflex stan (PMMA materials from the supplier Contaflex) to be 12.9 J/${\mathrm{cm}}^{2}$ and 13.9 J/${\mathrm{cm}}^{2},$ respectively, for a wavelength of λ = 1064 nm and pulse duration of τ = 10 ps. The difference in ablation threshold can be explained by the different pulse duration.

The ablation rate was determined for different pulse energies when the scanning velocity, the scanning pitch and the repetition rate were fixed to 3.6 m/s, 36.57 µm and 100 kHz, respectively. The maximum ablation rate was found to be 0.84 ${\mathrm{mm}}^{3}/s$ when the fluence is set to 78.5${\;J/\mathrm{cm}}^{2}$, which corresponds to an average laser power of 29 W, see Figure 4C. In this case it took approximately 1 min to ablate the 10 mm × 10 mm × 0.5 mm (50 mm^3^) shape. It is worth mentioning, that 50 mm^3^ is a common value which would be required to produce a custom 3D lens shape applicable for eye implants; therefore, it represents a realistic estimation of the time needed for the ablation task. After ablating the samples to a depth of 0.5 mm, one extra scan was performed with a low average power setting of 2.5 W to further decrease surface roughness. This way we were able to achieve the lowest surface roughness of Ra = 0.991 µm using the single pulse regime.

**Figure 5:**
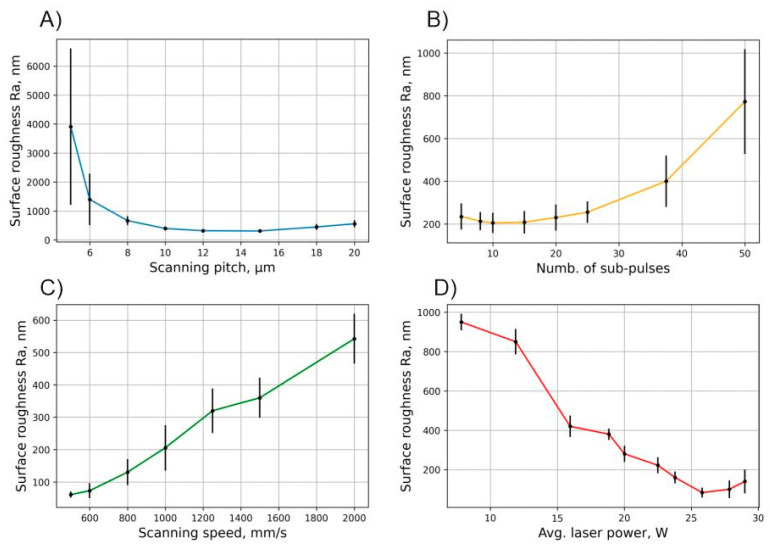
Surface roughness after polishing ablated samples dependent on different polishing parameters. Other fixed parameters in case of (**A**): P = 29 W, $N_{p}$ = 10 and v = 1000 mm/s; in case of (**B**): P = 19 W, dx = 10 μm and v = 1000 mm/s; in case of (**C**): P = 25.8 W, dx = 10 μm and $N_{p}$ = 10; and in case of (**D**): v = 1000 mm/s, dx = 10 μm and $N_{p}$ = 5.

**Figure 6:**
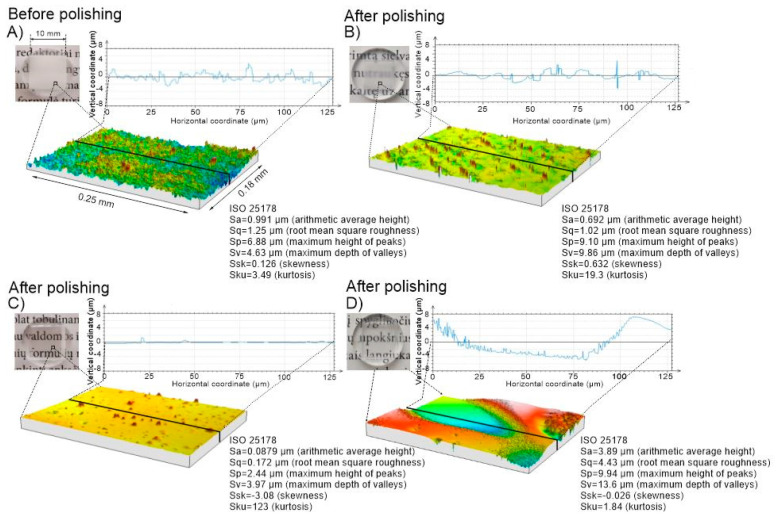
Visual representation of Ra (top left corner). Hydrophilic acrylic after ablation using parameters: P = 30 W, v = 3.65 m/s and dx = 36.5 µm + 2 additional scans with parameter set of P = 2.5 W, v = 1.03 m/s and dx = 10.3 µm (**A**). The same sample after polishing using the following parameters: P = 12 W, v = 1000 mm/s, dx = 5 μm and $N_{p}\;$ = 10 (**B**); after polishing with: P = 29 W, v = 1000 mm/s, dx = 10 μm and $N_{p}$ = 10 (**C**); and after polishing with P = 29 W, v = 1000 mm/s, dx = 5 μm and $N_{p}$ = 10 (**D**). Sa is a different notation of Ra, notation depends on ISO standard used.

The next step in lens production would be to initiate polishing of the produced samples by means of controlled surface melting using bursts of femtosecond pulses while using the same laser system setup. We have used bursts of femtosecond pulses where the sub-pulses had a temporal separation of 400 ps. From previous investigations [21] it was known, that the heat input on the surface would be much more pronounced in this time scale as compared to a larger temporal separation (nanoseconds) setting. However, it was unknown what the best parameter combination that produces the highest heat flux onto the surface is. In our experiments we varied the average laser power, the scanning speed, the scanning pitch and the number of sub-pulses within the burst and analyzed the roughness of the polished surface. The summarized results are shown in Figure 5. The results show that, under certain burst pulse parameter settings, it is possible to reduce the initial roughness of the sample made with the single pulse ablation mode. At 5 sub-pulses in the burst and low average power (~15 W) settings the achieved reduction in surface roughness is already about 2.5-fold, from ~1 μm to ~400 nm. We found that with higher average power settings (~24 W) it was possible to reach a surface roughness value below 100 nm (Ra). This result shows that depending on the scanning speed and spatial pitch settings a significant amount of laser power is required to be pointed onto the surface to initiate melting and surface smoothening. On the other hand, too much power can result in increase of surface roughness when the scanning speed is too high. As evident from the roughness dependence on the scanning speed graph, the surface roughness increases as the scanning speed increases, leading to insufficient thermal accumulation on the surface. However, it was noticed that if the thermal input is too large, boiling of the material can occur and large craters form on the surface producing a rise in the surface roughness as in the case of the low scanning pitch (5 µm) setting, see Figure 5A. It is likely, that the same boiling of the material will appear when the scanning speed would be decreased below the investigated range; however, since one of the goals is to produce rapid and high-quality polishing, low scanning speed settings were not investigated. The results suggest that depending on the used parameter configuration the surface quality after ablation can be enhanced up to >10-fold as can be seen in Figure 6C, or degraded by >3-fold, see Figure 6D. The transparency of the surface is dependent on the scattering properties of the surface. The Rayleigh roughness criterion R_at_ [37] may be used to evaluate the scattering characteristics of the surface when light is incident on the surface:

(1) $$R_{\mathrm{at}}{\;=\;k}_{0}S_{q}\frac{\left|n_{1}{\cos\theta }_{i}{-n}_{2}{\cos\theta }_{t}\right|}{2},$$

where k_0_—incident wave vector, S_q_—surface RMS roughness, n_1,2_—refractive indexes of incident mediums and θ_i,t_—incident and refracted. Generally, a surface may be regarded as flat with little to no scattering losses if R_at_ < π/16. Parts A and B appear opaque since R_at_ is higher by approximately an order of magnitude compared to the specified value in this case. Part C in Figure 6 is below the designated criterion and therefore appears transparent. An exception should be applied for part D where the surface appears transparent though shows large roughness values; this is due to the waviness that occurs on the surface when the hot bubbles explode upon reaching the surface. A wavy structure appears on the surface having smooth peaks and valleys. Such surfaces (part D) are not potential candidates for optical applications due to wavefront distortions that arise from the waviness. Overall, it was seen that the parameter space in which the surface roughness decreases drastically is relatively small and the best surface roughness of Ra = 60.8 nm was achieved using the following parameters: P = 25.8 W, v = 500 mm/s, dx = 10 µm and N_p_ = 10. This demonstrates that using these parameter settings we can achieve optimal thermodynamical flow, hence only melting the thin upper layer of the sample. Additionally, similar results (Ra = 83 nm) were also achieved using the following parameters: P = 25.8 W, v = 1000 mm/s, dx = 10 µm and N_p_ = 5. This indicates that it is possible to achieve surface smoothening even with a lower than 10 number of sub-pulses in the burst.

### 3.3. Investigation of Temperature Relation to Surface Roughness

**Figure 7:**
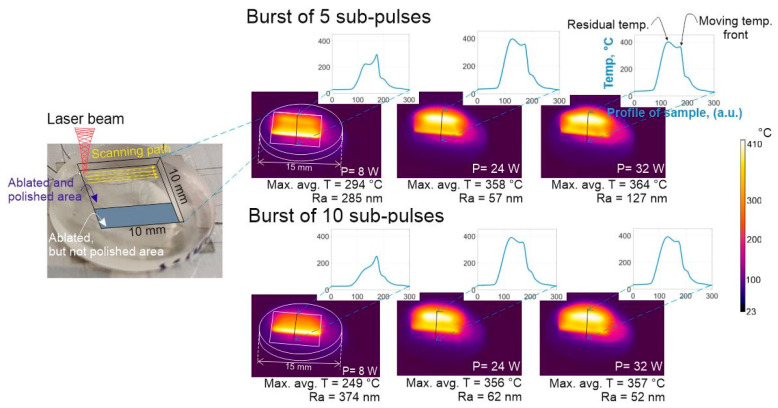
Temperature maps of the sample’s surface during micromachining and profiles of temperature maps averaged through the working area using different average laser power for bursts of 5 and 10 sub-pulses per burst. Max. avg. T listed below the images stands for maximum averaged temperature of the moving laser front. Other parameters used for polishing were: dx = 10 μm, v = 700 mm/s and number of scans = 2. Ra of the surface are partially dependent on the position where the measurement was performed, thus errors of ±25 nm must be taken into account.

From the previous section (see Figure 5C,D) it is evident that there are multiple micromachining parameter combinations that result in low (60.8 nm Ra) roughness values. The main cause for this is believed to be the rise in surface temperature up to a certain level to initiate melting of the rough regions. To investigate this further the temperature of the surface was recorded with an IR camera when performing the polishing procedure using different average power settings and number of sub-pulses within the burst. The 5 and 10 sub-pulses within the burst and the change of laser power from 8 to 32 W were chosen for comparison. The results are displayed in Figure 7. Correlation between the achieved surface roughness and surface temperature is evident (e.g., N_p_ = 5, P = 24 W and N_p_ = 10, P = 32 W). The measured surface roughness (57 nm and 52 nm, respectively) are excellent if maximum temperature is raised to around 357 °C. In this specific case a larger average power by two-fold is needed when using 10 sub-pulses within the burst as compared to 5 sub-pulses to achieve the best surface smoothness. This may be explained by taking into account that the absorption of light occurs via non-linear absorption processes, therefore more energy is absorbed within the material at lower average power settings when using 5 sub-pulses within the burst as compared to 10 sub-pulses, as the latter has a lower intensity. In addition, when performing the characterization of the bursts (see Figure 3) a trail of pre-pulses was registered for the 10 sub-pulse settings which was absent for the 5 sub-pulse case. How the pre-pulses affect the absorption of light and the polishing process is beyond the scope of this article, though it remains an interesting topic for future research. It was noticed that, during laser polishing when the temperature of the surface was higher than 300 °C, a high accumulated temperature zone formed (see Figure 7, residual heat) at the edge of the rectangle where the beam scanning started, in other words, the zone which is always opposite to the moving heat front and is maintained until the end of the polishing procedure. The moving temperature front is associated with the polishing beam position; as the photographs presented in Figure 7 are taken during the polishing process therefore the moving temperature front is visible. A possible explanation regarding the origins of the accumulation of temperature in the residual heat zone is the reflection of light from the slope of the laser impingement area (Figure 2, “melt pool”) at the point where the molted material forms. The laser light that was reflected impinges the edge of the cuboid and increases the temperature slightly during the polishing process. Besides that, it restricts the area from cooling down via diffusion of heat. The molten material can exhibit optical quality properties and thus reflects light well. In addition, it is known [38] that a plasma cloud forms after the first pulse has impinged on the surface, and does not dissipate until the second pulse (after 400 ps) arrives at the surface. Depending on multiple machining conditions, a portion of light can also reflect from the plasma cloud. However, the stated assumptions need to be proven experimentally and remain a topic of future research.

The minimal surface roughness achieved after a single scan over the surface was ~250 nm. It is known that for metals, scanning the surface multiple times increases the smoothness of the surface [39,40]. Therefore, the surface roughness was also measured when performing several scans (a change in the scanning angle by 90° is produced after each pass). It was noticed that scanning twice improves the surface finish as compared to the single scan case; however, when the scanning was done more than twice the surface roughness increases again and the sample becomes non-transparent. This may be explained by noting that after a number of scans, a deeper portion of the material reaches melting point and the material starts boiling. Bubbles rise to the surface and leave large craters as can be seen in Figure 6D. Therefore it is likely that the achieved minimal surface roughness value (Ra = 18 nm using parameters: P = 22 W, N_p_ = 5, v = 620 mm/s, dx = 10 µm, number of scans = 2 and f = 100 kHz) cannot be reduced further due to the Marangoni effect [41].

**Figure 8:**
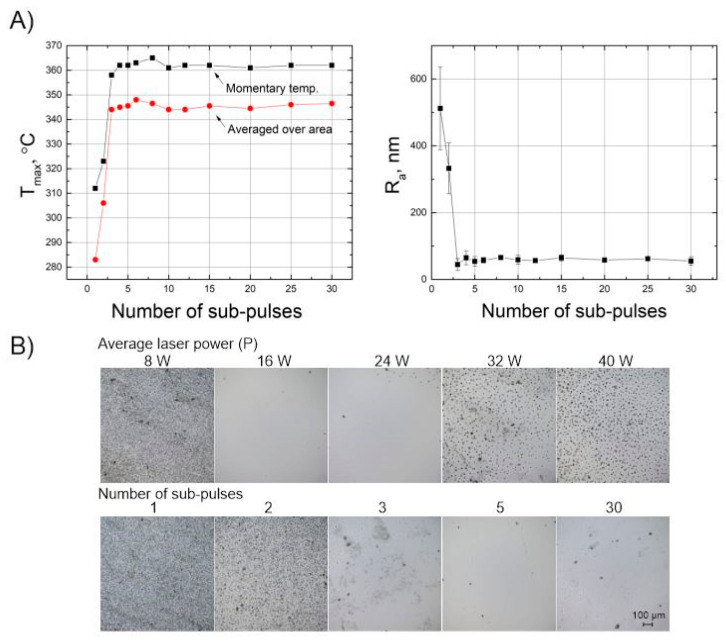
Surface temperature dependence on the number of sub-pulses within a burst (left picture), surface roughness dependence on the number of sub-pulses within a burst (right picture). Other parameters: P = 16 W, dx = 10 µm, v = 700 mm/s and number of scans = 2 (**A**). The surface of the sample after polishing using different average laser power when other parameters are $N_{p}$ = 10, dx = 10 µm, v = 700 mm/s and number of scans = 2 (**B**, first line). The surface of the sample after polishing using different numbers of sub-pulses when other parameters are P = 16 W, dx = 10 µm, v = 700 mm/s and number of scans = 2 (**B**, second line).

Figure 5 showed that by using bursts of femtosecond laser pulses it was possible to increase the surface quality. To further examine how the number of sub-pulses in the burst affects the polishing quality, we looked into how the number of sub-pulses within the burst correlates to the sample’s surface temperature. Figure 8A shows that when the number of sub-pulses rises from 1 to 3, the surface temperature increases from 285 to 355 °C, while the roughness of the samples decreases to below 100 nm. Although, increase of the surface quality is only apparent until a single pulse is split into three, further increasing the number beyond this value does not increase the surface quality. These observations suggest that when splitting a single pulse into three sub-pulses, a certain energy threshold per pulse is reached and thermodynamical processes are stimulated which result in the remelting of the surface and its smoothening. Furthermore, Figure 5D shows that the temperature dependence on the average laser power is not linear. We have noticed that when increasing the average power of the laser, the temperature rises quickly to 350–360 °C. When the surface temperature increases to more than 360 °C, bubbles form below the surface. The bubbles are the release of gas trapped inside the volume which explode upon reaching the surface, this causes sputtering of the molten material and creates micro-valleys. In turn, this causes an increase in the surface roughness [42]. These experiments show, that the optimal temperature at which the best surface quality can be achieved is approximately 357 °C. The images of the surface are depicted in Figure 8B, and that no recognizable difference is seen for the case of 3–30 sub-pulses per burst.

**Figure 9:**
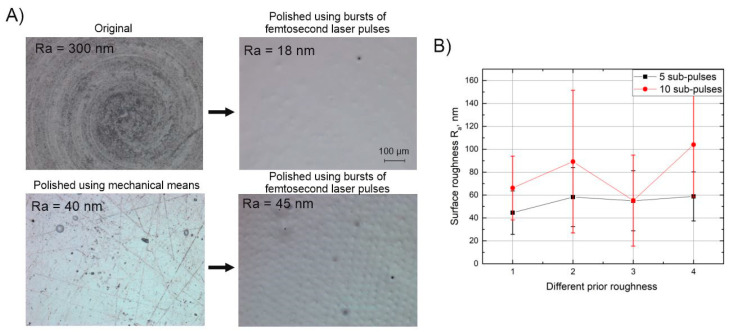
Pre-polished and original samples after polishing using bursts of femtosecond laser pulses (**A**), surface roughness after polishing using bursts of femtosecond laser pulses dependent on the different prior roughnesses when $N_{p}\;$= 5 and $N_{p}\;$ = 20 (**B**). Parameters used in (**A**) section are: P = 22 W, $N_{p}\;$ = 5, v = 620 mm/s, dx = 10 µm, f = 100 kHz and number of scans = 2. Parameters used in (**B**) section are: P = 32 W, v = 700 mm/s, dx = 10 µm, f = 100 kHz and number of scans = 2.

Since it is thought that the Marangoni effect is the limiting factor preventing further decease in the surface roughness, experiments were performed investigating the relation between the surface roughness before polishing (prior roughness) and the surface roughness after polishing (final roughness). Initially, it was expected that larger differences would be present when performing the polishing procedure on different roughness surfaces. The experiment was carried out by preparing a number of samples, each having different initial roughness values ranging from 0.75 to 3 µm Ra. Different surface roughness values were achieved by varying the average power of the laser without using burst-mode. The 3 µm surface roughness value was achieved by adjusting the spatial pitch of the hatching lines to dx < 5 µm. In addition, for comparison purposes, a number of samples were polished mechanically to a roughness of 40 nm Ra. After polishing the different roughness samples no significant difference in the final roughness was detected when comparing polishing with 5 and 10 sub-pulses in the burst, see Figure 9B. The results fall within an error range of one standard deviation of 50 nm. This result again confirms that as long as a certain temperature is reached on the surface during the polishing step, the results do not differ even when changing the number of sub-pulses from 5 to 20. In addition, it shows that the surfaces with roughness in range of 0.75–3 µm can be successfully polished to the same finish level using method presented in this study. However, for the case of the mechanically polished samples, further reduction of the surface roughness was not achieved. As visible in Figure 9A, small artifacts resembling dimples are present on the surface of sample after polishing with bursts, which contribute to a surface roughness value of 45 nm Ra. These artifacts are thought to be the result of embedded polishing particles in the surface, a residual from mechanical pre-polishing. Eventually, best surface quality was achieved by polishing via laser prepared samples with parameters: P = 22 W, N_p_ = 5, v = 620 mm/s, dx = 10 µm, f = 100 kHz and scanning twice. With the former parameter configuration we were able to achieve ~20 nm Ra and ~30 nm Sq, which according to Rayleigh roughness criteria R_at_ = 0.1 < π/16 represents a flat surface with little to zero scattering losses.

**Figure 10:**
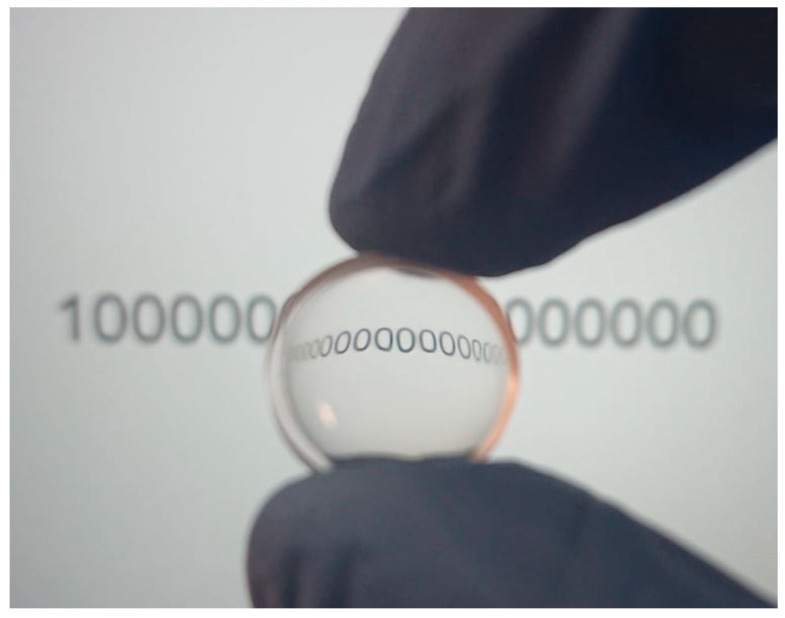
Polished curved surface. Polishing parameters: P = 22 W, $N_{p}\;$= 5, v = 620 mm/s and dx = 10 µm.

For demonstration purposes, a concave lens of 13.5 mm diameter and having a radius of curvature of 20.5 mm was fabricated. The shape was ablated using parameters: v = 2500 mm/s, P = 16 W, dx = 20 µm and polished with following parameter set: P = 22 W, N_p_ = 5, v = 620 mm/s, dx = 10 µm and f = 100 kHz. The result is shown in Figure 10. It is evident that the fabricated concave lens is fully transparent, smooth and bends the image like a lens.

## 4. Conclusions

In this study we have investigated the ability to polish the hydrophilic acrylic polymer Contamac CONTAFLEX 26% UV-IOL (R), which is used as a bio-compatible polymer for body implants manufacturing, using bursts of femtosecond laser pulses. The parametric study of the laser machining parameters that consist of the following micromachining parameters: average laser power, scanning speed, scanning pitch and number of sub-pulses within the burst, showed that polishing up to a surface roughness value of Ra = 40 nm is achievable. The analysis of the surface temperature during micromachining showed that the polishing process depends on the thermal heat flux which initiates thermodynamical processes that change the surface roughness of the sample. The optimal surface temperature was found to be approximately 357 °C, at which it was possible to polish the material up to 18 nm Ra, resembling a transparent surface which meets optical-quality standards. The correlation between the temperature at the surface and the achieved surface roughness values is presented which shows that such roughness values are achievable through variation of the number of sub-pulses within the burst. In addition, it was shown that in order to reach the stated temperature levels, it is necessary to split the single pulse into at least more than three sub-pulses. However, if the number of sub-pulses is increased above three, similar results in terms of surface temperature may be achieved. If the surface is overheated to above 360 °C, micro bubbles form which rise to the surface and produce surface craters upon exploding, dramatically reducing the surface quality. It was shown that the burst-pulse polishing process is invariant of the initial roughness of the surface in the range of 0.7–3 µm Ra. In addition, if the surface has embedded mechanical polishing artifacts (abrasive particles), the surface degrades after the polishing procedure. Finally, it was shown that results achieved in this study can be applied to manufacturing of free-form optical components such as lenses, providing a fast (few minutes per lens) and efficient way of polishing to achieve low roughness surfaces.
